# Outcomes of coblation tonsillectomy versus bipolar electrocautery tonsillectomy in pediatric population

**DOI:** 10.1186/s43163-022-00340-9

**Published:** 2022-11-26

**Authors:** Ahmad M. Eltelety, Mohamed E. Swelam, Hazem M. Dewidar, Ahmed M. El Batawi

**Affiliations:** 1 Otolaryngology Department, Faculty of Medicine, Al Kasr Al Ainy School of Medicine, Cairo University, Manial, Cairo, 11451 Egypt; 2 Otolaryngology Department, Agouza Police Hospital, Ministry of Interior, Giza, Egypt

**Keywords:** Tonsillitis, Tonsillectomy, Bipolar electrocautery, Coblation, Healing, Bleeding, Pain

## Abstract

**Background:**

Bipolar electrocautery tonsillectomy has been the preferred technique for many otolaryngologists, yet coblation tonsillectomy is gaining popularity in the current practice. This study aims at comparing both techniques in terms of pain, bleeding, and healing.

**Results:**

A total of 120 patients were randomly divided into two equal groups. Overall mean pain score associated with coblation tonsillectomy was statistically less than that caused by bipolar electrocautery throughout the follow-up period (*p* < 0.001). The difference in pain duration was statistically longer for the bipolar group. The incidence of postoperative hemorrhage—both reactionary and secondary—was statistically higher in the bipolar group. Coblation tonsillectomy showed statistically shorter duration of healing (*p* < 0.001).

**Conclusions:**

Coblation tonsillectomy is associated with less pain severity and shorter pain duration, fewer bleeding incidents, and more prompt healing.

## Background

Tonsillectomy is the most common surgical procedure in the setting of pediatric otolaryngology practice. The procedure is mainly carried out to treat disorders of sleep and recurrent attacks of acute tonsillitis; however, these are not the only indications [1]. The indications adopted by the American Academy of Otolaryngology – Head and Neck Surgery (AAO-HNS) are the most widely utilized guidelines in the current practice [2].

Tonsillectomy is generally divided into two main surgical techniques: the complete tonsillectomy (capsular) technique and the partial tonsillectomy (intracapsular tonsillotomy technique). With capsular dissection, the tonsil is grasped medially, and the dissection starts lateral to the tonsil and medial to the superior constrictor muscle. Among the surgeon’s armamentarium for the dissection are cold steel instruments and suture ligatures, unipolar electrocautery, bipolar electrocautery, coblation wands, harmonic scalpel, and CO^2^ laser [3]. Cold steel tonsillectomy entails dissection with scissors usually from cranial to caudal direction till the lower pole which is usually controlled—together with other bleeding points—by ties. Electrocautery dissection provides the simultaneous privilege of both dissection and hemostasis (whether unipolar or bipolar). With the use of the bipolar electrocautery technique, electricity is trapped between the two tips of the instruments without being disseminated throughout the body, and hence, it is safer to use in patients with implantable electrical devices like cardiac pacemakers [4]. The harmonic scalpel (Ethicon Endo-Surgery Inc., Cincinnati, OH) has been proposed as one of the tools available for tonsillectomy. It utilizes ultrasonic shears with frequency of 55,000 cycles per second. The resultant energy provides both cutting and sealing of the tissues with minimal thermal collateral damage. The high cost remains a challenge against the wide use of this technique [3]. Intracapsular tonsillotomy has been proposed by some surgeons with debatable safety profile and questionable recurrence rates [5]. The most commonly used techniques for intracapsular tonsillotomy are microdebrider, bipolar radiofrequency ablation, and CO^2^ Swiflase cryptolysis [3, 6]. The microdebrider is commonly used for intracapsular tonsillotomy. The technique depends mainly on a rotatory shaving device that is continuously connected to a suction system that pulls and hence cuts the tissues. Radiofrequency ablation of the tonsils results in ionization of a conductive saline medium which generates a molecular dissection energy that ablates the tonsillar tissues. This is also known as coblation low-temperature plasma excision. The later technique can be used for both tonsillectomy and tonsillotomy [3]. Both tonsillectomy and tonsillotomy are acceptable treatment options for pediatric patients with sleep-disordered breathing; however, only complete tonsillectomy is accepted for patients undergoing tonsillectomy due to chronic tonsillitis [3, 4].

A systematic review of the Cochrane database has shown a trend towards less pain with the use of the coblation over other tonsillectomy techniques (including both hot and cold techniques) on postoperative days 1 and 3 but not 7; however, these findings are still debatable, and their clinical significance remains challenged due to lack of research in this arena. When bleeding was studied as an outcome, intraoperative bleeding was reported to be less in the coblation over other techniques, but the risk of secondary hemorrhage seemed to be higher with the use of coblation. Again, these findings lacked the clinical significance and failed to gain the confidence of the authors to suggest the superiority of coblation over other techniques due to lack of sufficient properly designed literature [7].

Bipolar electrocautery tonsillectomy has been widely used in everyday surgical practice. More recently, coblation tonsillectomy has been gaining popularity with rising evidence of comparable results to both standard cold steel tonsillectomy and bipolar electrocautery tonsillectomy [8, 9]. The main outcomes of clinical concern after tonsillectomy are pain duration and severity, postoperative bleeding, and duration of the recovery process [10].

The aim of this study is to compare between the clinical outcomes after bipolar electrocautery tonsillectomy versus coblation tonsillectomy in pediatric population after a week, 2 weeks, a month, and 3 months with regards to pain severity and duration, bleeding, healing and assessment of the complete separation of white healing membrane of the operative bed.

## Methods

This is a prospective randomized controlled study. It included 120 patients. They were randomly divided into two equal groups; the first group included patients who underwent coblation tonsillectomy (group A), and the other group included patients who underwent bipolar electrocautery tonsillectomy (group B). Patients were recruited from the otolaryngology outpatient clinic at a tertiary care institution during the period between February and August 2022. All patients and their guardians gave their consent to participate in the study and to complete the follow-up sheets regarding the clinical data of the patients. This study was approved by the research ethics committee (REC) under the approval number of: MS-606-2021.

### Inclusion criteria

1. Patients’ age between 3 and 12 years old (both genders) complaining of recurrent attacks of acute follicular tonsillitis; according to the AAO-HNS [2]
2. Patients complaining of snoring and/or sleep apnea
3. Associated enlarged adenoid will be included as well

### Exclusion criteria

1. Patients with metabolic syndromes or storage diseases
2. Patients with Down syndrome
3. Bleeding tendencies
4. Patients below 3 or over 12 years old
5. Patients lost to follow-up or guardians refusing to participate in the study

### Method of randomization

In the clinic, the guardians were offered two upside-down cards; each contained a letter (A or B). Each letter contained the name of the technique they would undergo (A = coblation, B = bipolar). Approving guardians chose a card randomly.

### Pre-procedural assessment

Both groups were subjected to the same preoperative assessment measures including detailed history taking, complete physical examination and routine preoperative investigations.

### Operative procedure

**Fig. 1:**
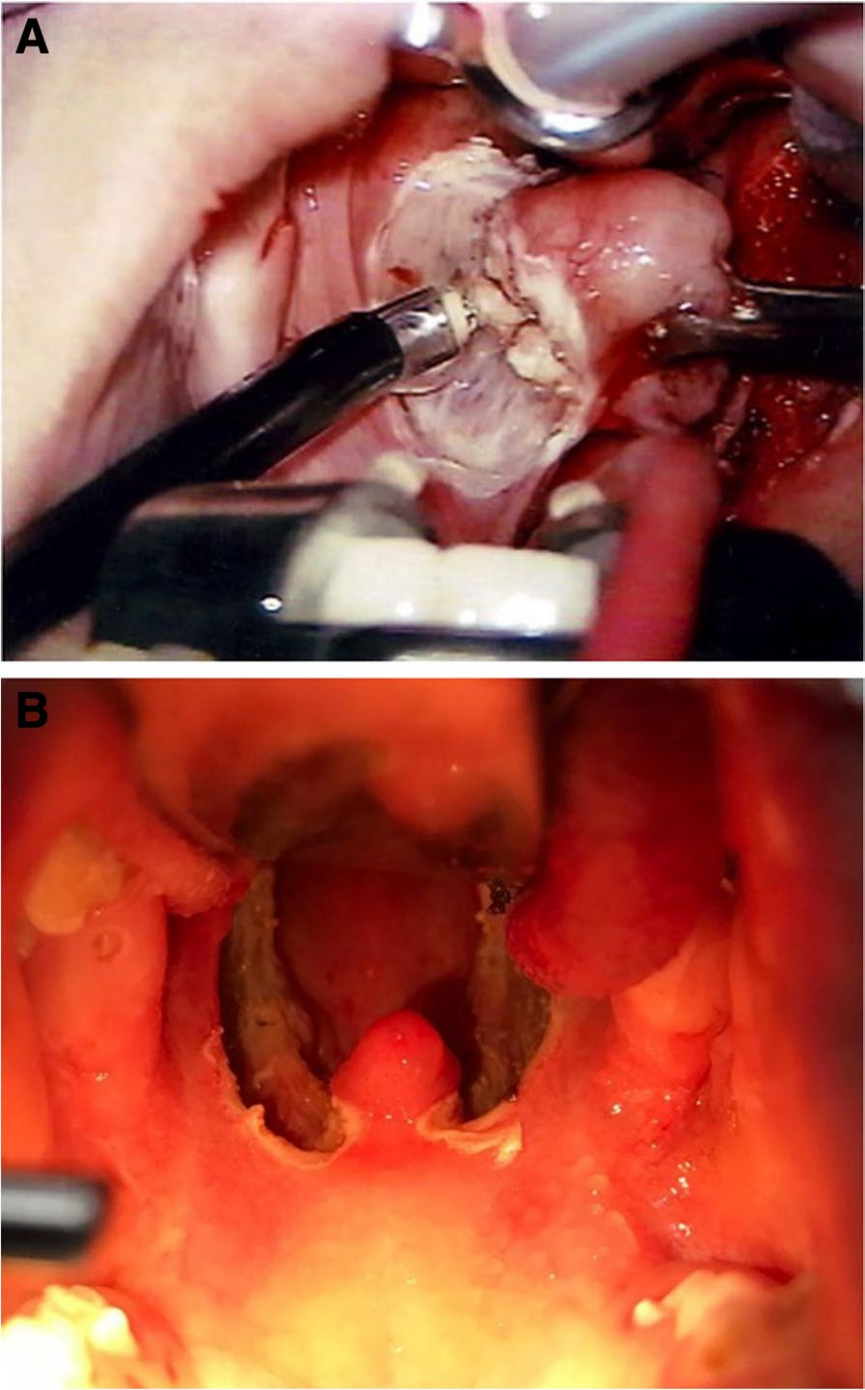
**A** Intraoperative image of coblation tonsillectomy. **B** Immediate postoperative image of the surgical bed in coblation tonsillectomy

**Fig. 2:**
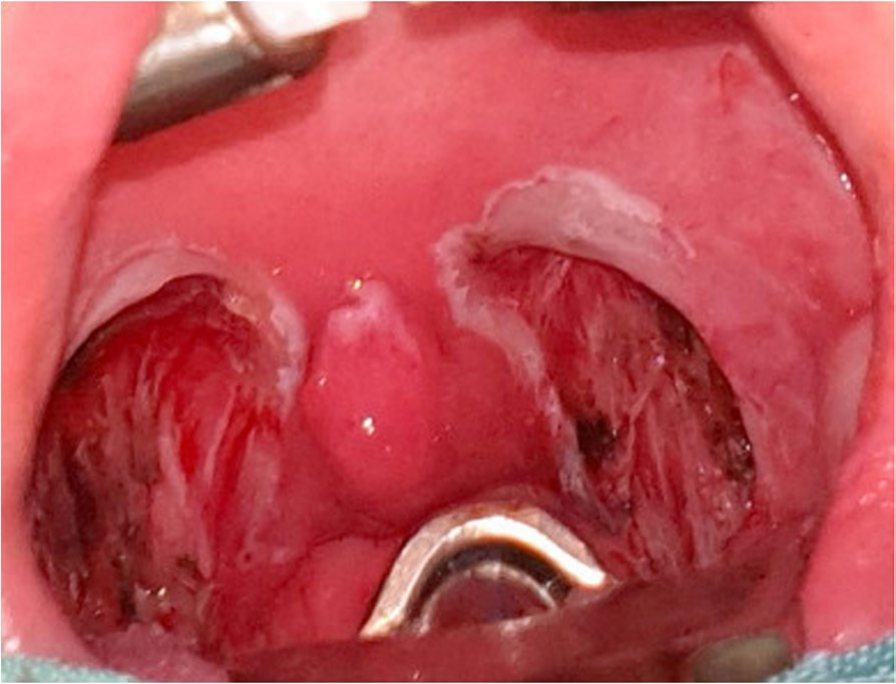
Immediate postoperative image of the surgical bed in bipolar tonsillectomy

There were 2 groups, each of which underwent surgery by a different technique:

- Group A (coblation tonsillectomy group): 60 patients were included using *Evac 70 Xtra Plasma Wand (Arthrocare, Sunnyvale, California)*. An ablation setting of 7 for the tonsils removal and coagulation setting of 3 were used for all patients in this group (Fig. 1).
- Group B (bipolar electrocautery tonsillectomy group): 60 patients were included (*using Coviden Force FX with straight bipolar forceps*). The device was set for Med power (standard setting) (40) for both dissection and cauterization (Fig. 2).
- All patients had the procedure done under general anesthesia and oral endotracheal intubation.

### Postoperative care

All patients were subjected to the same following measures. Acetaminophen and ibuprofen were used for pain control. Antibiotics were given in the form of amoxicillin/clavulanate or clarithromycin in case of penicillin allergy.

After surgery, all patients were followed up on fixed intervals a week, 2 weeks, a month, and 3 months after surgery regarding bleeding, pain severity and duration, and healing (complete separation of the whitish healing membrane). The guardians answered the inquiries in the sheet, and complete physical examinations were conducted on each visit.

No postoperative images of the surgical bed were taken during the follow-up visits to avoid disturbing the delicate pediatric patients of our study population. On-site assessment was the source of information.

### Study outcomes

**Fig. 3:**
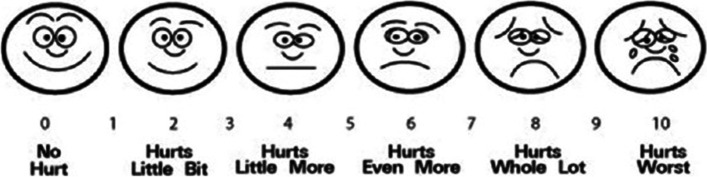
Wong-Baker FACES pain score

1. Pain: using Wong-Baker FACES pain score (Fig. 3) [11].

On each visit, the guardian chose the suitable picture that expressed the patient mood and pain level, and then the pain scale—between 0 and 10—would be scored according to guardian’s choice. On the last visit, the overall pain scale was scored over 40 (the total number of 4 visits). A score of 40 represented the worst pain scale. The duration of pain was also recorded as well.

2. Postoperative bleeding (reactionary and secondary): the incidence of bleeding incidents was recorded for both groups.
3. Healing (complete disappearance of the white healing membrane): each time, the tonsillar bed was checked for healing and the disappearance of the white healing membrane. The guardians were informed on the appearance of the whitish membrane and were also instructed to perform daily monitoring of their patients. The exact day of complete disappearance of the white membrane was determined accordingly.

### Statistical analysis

The averages per patient over time were used as the main outcome measures using the Wilcoxon signed-rank test and Wong-Baker FACES rating scale for each value and each group. Data were coded and entered using the Statistical Package for the Social Sciences (SPSS) version 28 (IBM Corp., Armonk, NY, USA). Data was summarized using mean and standard deviation. Comparisons between groups were done using unpaired *t*-test. *P*-values less than 0.05 were considered as statistically significant. Student’s *t*-test was used to compare between two means. Again, as these measures were taken over time, a repeated measures analysis was used to assess the correlation of these items over time.

## Results

This study included 120 patients divided into 2 equal groups. Group A included patients who underwent coblation tonsillectomy, and group B included patients who underwent bipolar electrocautery tonsillectomy. Data assessed included age, sex, postoperative pain severity and duration, postoperative bleeding, and healing at 1st week, 2nd week, 1st month, and 3rd month after the procedure.

1. Comparison between both groups regarding patients’ demographics:

The mean age of the study population was 7 years. Both groups showed similar demographic distribution without statistical significance (Table 1).

2. Comparison between both groups regarding postoperative pain severity and duration (Tables 2 and 3):

**Table 1:** Comparison between both groups regarding patients’ demographics

	Group A (***n*** = 60)	Group B (***n*** = 60)	***P***-value
**Age**			0.126 **(non-significant)**
**Range**	3–12	3–12	
**Mean ± SD**	7.7 ± 2.9	6.9 ± 3	
**Gender**			0.143 **(non-significant)**
**Male**	27 (45%)	36 (60%)	
**Female**	33 (55%)	24 (40%)

**Table 2:** Comparison between mean pain scores for both groups on each individual visit

	Groups A (mean ± standard deviation)	Group B (mean ± standard deviation)	***P***-value
**Mean pain score on 1st visit**	9.32 ± 0.62	9.97 ± 0.18	0.001 **(significant)**
**Mean pain score on 2nd visit**	5.62 ± 1.19	9.03 ± 0.71	0.001 **(significant)**
**Mean pain score on 3rd visit**	2.10 ± 1.24	6.13 ± 1.91	0.001 **(significant)**
**Mean pain score on 4th visit**	0.22 ± 0.42	1.75 ± 1.90	0.001 **(significant)**

**Table 3:** Comparison between both groups for different study outcomes

	Group A (***n*** = 60)	Group B (***n*** = 60)	***P*** value
**Overall mean pain scores**			**0.001 (significant)**
**Range (0–40)**	13–22	20–35	
**Mean ± standard deviation**	17.2 ± 2.6	26.9 ± 4.1	
**Pain duration in days**			**0.001 (significant)**
**Range**	13–21	21–35	
**Mean ± standard deviation**	16 ± 2.2	25.3 ± 3.7	
**Reactionary and secondary bleeding**			**0.027 (significant)**
**Yes**	0 (0%)	6 (10%)	
**No**	60 (100%)	54 (90%)	
**Healing/day**			**0.001 (significant)**
**Range**	8–17	15–26	
**Mean ± standard deviation**	13.3 ± 2.1	20 ± 3.5	

According to Wong-Backer FACES score, pain score for each visit in group A (coblation) ranged between (8–10), (3–8), (0–5), and (0–1) with mean pain scores (mean ± standard deviation): 9.32 ± 0.62, 5.62 ± 1.19, 2.10 ± 1.24, and 0.22 ± 0.42 on the 1st, 2nd, 3rd, and 4th visits respectively. In group B (bipolar), the score for each visit ranged between (9–10), (7–10), (2–9), and (0–6) with mean pain scores (mean ± standard deviation): 9.97 ± 0.18, 9.03 ± 0.71, 6.13 ± 1.91, and 1.75 ± 1.90 on 1st, 2nd, 3rd, and 4th visits, respectively. Mean pain score on each interval in group A was less than group B with a significance level of (*P*-value = 0.001). The overall mean pain score was significantly less in group A (*P*= 0.0001), while the duration of pain was significantly longer in group B (*P*-value = 0.0001).

3. Comparison between both groups regarding the incidence of postoperative bleeding:

In group A, there were no cases of reactionary or secondary bleeding. In group B, there were 6 cases of postoperative hemorrhage. Two patients bled after 24 h (reactionary), two after 48 h (reactionary), one after 4 days (secondary), and one after 1 week (secondary). All of them were admitted to the hospital, and the bleeding was controlled by conservative methods. None of them needed reoperation.

4. Comparison between both groups regarding the duration of healing as indicated by the complete disappearance of the whitish healing membrane: the duration of healing for patients in group B was significantly longer than those in group A (*P*-value = 0.0001). Table 3 exhibits detailed discussion on different study outcomes.

## Discussion

Our current report aimed at comparing the outcome of coblation versus bipolar electrocautery tonsillectomy. The outcome parameters were the occurrence of postoperative bleeding, duration of healing (as indicated by the complete disappearance of the white healing membrane), postoperative pain severity, and pain duration.

The use of coblation in surgical practice is mainly dependent on chemical rather than thermal effect. This principle paved the way for the safe use of coblation during tonsillectomy procedures to reduce postoperative pain severity and duration [12].

In the current report, the difference in pain severity between both groups was statistically significant on each of the follow-up visits; however, on the first visit, the difference in the mean pain scores did not reflect a meaningful clinical difference. The authors have also found that the overall mean pain score and pain duration were significantly better for the coblation group. This comes in agreement with what has been previously reported by Omrani and associates [13]. Polites and associates agreed to the better performance of coblation over dissection tonsillectomy during the first 3 days only. Subsequent evaluation did not show statistical differences [14]. The superiority of coblation tonsillectomy over blunt dissection tonsillectomy was advocated in a report by Martin and associates in a randomized prospective study including 200 patients [15].

These findings were opposed by the results reported by Hasan et al [8]. This disagreement maybe explained by the difference in the sample size between both studies. We were able to recruit a total of 120 patients, while Hasan and associates included 80 patients in their study. Coblation tonsillectomy was compared to monopolar electrocautery tonsillectomy in a study reported by Jones and colleagues [16]. In their report, the authors concluded that coblation tonsillectomy was superior to monopolar electrocautery tonsillectomy only on the day of surgery. This difference was of limited clinical use when weighted against the higher cost of the coblation device. It is not to be neglected that they included a relatively small sample size and used both techniques for the same patient with one side acting as the control while the contralateral side resembled the interventional side. As well, they included patients with age up to 21 which is different from our study population.

In the present study, it was found that coblation tonsillectomy had better performance than bipolar tonsillectomy in terms of postoperative hemorrhage. The bipolar group had 4 patients who developed reactionary hemorrhage and two who suffered from secondary bleeding. No cases in the coblation group had any postoperative bleeding events. This difference was both statistically and clinically significant. A study by Belloso and associates agreed with our report with emphasis that this difference in postoperative hemorrhage was especially significant for the pediatric population [9]. Another study by Omrani et al showed modest results with a trend towards the superiority of coblation tonsillectomy over traditional surgical techniques; however, this difference did not reach statistical significance [13]. A meta-analysis of the published literature agreed that coblation tonsillectomy showed safe and acceptable results—similar to the standard tonsillectomy techniques—in terms of postoperative bleeding [17]. This finding was proposed by another study from Denmark. In their report, the authors conducted a non-blinded prospective study which included population different from ours [18].

The difference in the incidence of postoperative bleeding events in our study can be explained by the fact that coblation tonsillectomy produced less pain with improved swallowing ability and better oral intake in the early postoperative period. This privilege has its impact on the reduction of operative bed infection which can lead to serious hemorrhagic consequences.

In our study, the healing of the operative bed was more prompt for the coblation group. This is indicated by the complete disintegration of the whitish healing membrane and complete epithelialization of the operative bed. This advantage was statistically significant when compared against the bipolar group. This comes in agreement with what has been reported by Timms and Temple in 2002 [19] and by Matin and associates [15]. Temple and Timms agreed to our finding in their report regarding the faster healing in the coblation group. In their report, all patient showed complete sloughing of the healing membrane during the second week after the procedure [20].

In contradiction to this finding, a study by Rakesh and associates found that healing was significantly longer on the coblation group up to 1 week after the procedure. Follow-up after 3 weeks showed no difference between coblation versus dissection tonsillectomy [21].

The faster healing offered by coblation tonsillectomy is best explained by the fact that coblation produces less thermal heat during the dissection and hence less tissue trauma. This leads to faster recovery, less granulation tissue formation, and less scarring. Coblation device usually produces heat between 45 and 85 °C, while bipolar devices usually produces head around 300 and 400 °C [12, 22].

This study was affected by the effect of the outliers on the means of the outcomes. The mean tends to skew towards the outlier. To preserve the integrity of the data in this study, outliers were included and not excluded despite the fact that this may lead to contamination of the results [23]. In the present report, the mean duration of pain in the bipolar group was 25 days. This is attributed to the fact that only one patient had pain up to the 35th day postoperatively. Despite being an odd finding for the post-tonsillectomy pain to extend this long, however, this can be explained by possible personal variations between individuals with different tolerability to pain and pain threshold levels. Also, the guardians were the main source of reporting the pain indices for their children. There might be misinterpretation or exacerbation by the guardians for the duration of pain expressed by their children. Pediatric patients can also misinterpret temporomandibular joint related pain as pain related to their throat especially that both conditions are related to eating. Another example of outliers affecting our results is the presence of one patient in the coblation group with complete separation of the healing membrane on postoperative day 8 with the mean duration for complete healing in this group being 13 days. This relatively early duration of healing (postoperative day 8) may be explained by the fact that assessment of the parents was the main source for reporting the outcome in the intervals between the follow-up visits.

The main strength of this study is stemmed from its prospective nature. Effective randomization adds to its strength. The duration of the follow-up period is not only sufficient to judge early outcomes like pain severity but is also adequate to evaluate postoperative pain duration, postoperative hemorrhage, and healing of the operative bed.

The main drawback of this study is the relatively small sample size. This can be explained by the timeframe of the conduction of the study which was during the COVID-19 pandemic. Major healthcare resources were mainly directed to isolation and advanced care facilities. Many patients willingly agreed to postpone elective procedures to further save more resources to counteract the pandemic. Also, the relatively short duration of the study—7 months—have also contributed to this shortage in the number of patients. Another drawback is the method of assessment of healing, which was mainly dependent on the guardians’ observations of the white healing membrane. This is also explained by the fact that identifying the exact date of complete separation of the membrane needed examination of the patients by the surgeon every day, something that is practically unfeasible. For this, the researchers had to educate the guardians of the shape and characteristics of the white healing membrane and gave them information and training on each visit on how to observe its existence and to accurately document its disappearance. Another drawback is the lack of proper matching of the indication for tonsillectomy for both groups. This drawback also entails the lack for control for both groups regarding the extent of the procedure, i.e., whether tonsillectomy was performed alone or in combination with adenoidectomy. It is to be emphasized that usually for younger pediatric population, the procedure of tonsillectomy is usually coupled with concurrent adenoidectomy. In the current report, most of the patients had simultaneous tonsillectomy and adenoidectomy. As well, the majority of patients in our study had recurrent throat infections rather that sleep disordered breathing as the indication for the procedure. In order to confirm the diagnosis of obstructive sleep apnea, a polysomnogram is needed. In fact, the medical literature recommends against the routine use of polysomnography except in patients with syndromes affecting their craniofacial skeleton [24]. The disagreement in recommendations by three American medical societies reflects the discordance regarding the applicability of sleep studies in clinical practice [23]. Most of these patients were already excluded from our study, and hence, the guardians’ history was the source for suspecting the diagnosis of sleep-disordered breathing rather than any other tests. The relatively high cost of any investigative procedures - to confirm the diagnosis of obstructive sleep apnea - and the cultural difficulties added to the hurdles of routine testing for this indication prior to commencing on the study.

## Conclusion

Our study indicates that coblation tonsillectomy is superior to bipolar tonsillectomy with fewer postoperative bleeding incidents, shorter recovery, less pain severity scores, and shorter pain duration. This difference is of limited clinical value during the first week after surgery. These benefits should be carefully weighed against the higher cost of the coblation device.

## Data Availability

The datasets used and/or analyzed during the current study are available from the corresponding author on reasonable request.
